# Homozygous mutation of *VPS16* gene is responsible for an autosomal recessive adolescent-onset primary dystonia

**DOI:** 10.1038/srep25834

**Published:** 2016-05-12

**Authors:** Xiaodong Cai, Xin Chen, Song Wu, Wenlan Liu, Xiejun Zhang, Doudou Zhang, Sijie He, Bo Wang, Mali Zhang, Yuan Zhang, Zongyang Li, Kun Luo, Zhiming Cai, Weiping Li

**Affiliations:** 1 Department of Neurosurgery, Shenzhen Second People’s Hospital, Shenzhen University 1^st^ Affiliated Hospital, Shenzhen, Guangdong 518035, China; 2 Department of Urology, Sun Yat-sen University Cancer Center; State Key Laboratory of Oncology in South China; Collaborative Innovation Center for Cancer Medicine, Guangzhou 510060, China; 3 BGI Cognitive Genomics Lab, Building No. 11, Beishan Industrial Zone, Yantian District, Shenzhen, Guangdong 518083, China; 4 Collaborative Innovation Center for Cancer Medicine National-Regional Key Technology Engineering Laboratory for Clinical Application of Cancer Genomics, Shenzhen Second People’s Hospital, Shenzhen University 1stAffiliated Hospital, Shenzhen, Guangdong 518035, China; 5 The Central Laboratory, Shenzhen Second People’s Hospital, Shenzhen University 1^st^ Affiliated Hospital, Shenzhen, Guangdong 518035, China; 6 The First Clinical College of Anhui Medical University, Hefei 230032, China; 7 School of Medicine, Shandong University, 44 West Culture Road, Ji’nan, Shandong 250012, China

## Abstract

Dystonia is a neurological movement disorder that is clinically and genetically heterogeneous. Herein, we report the identification a novel homozygous missense mutation, c.156 C > A in *VPS16*, co-segregating with disease status in a Chinese consanguineous family with adolescent-onset primary dystonia by whole exome sequencing and homozygosity mapping. To assess the biological role of c.156 C > A homozygous mutation of *VPS16*, we generated mice with targeted mutation site of *Vps16* through CRISPR-Cas9 genome-editing approach. *Vps16* c.156 C > A homozygous mutant mice exhibited significantly impaired motor function, suggesting that *VPS16* is a new causative gene for adolescent-onset primary dystonia.

Dystonia is characterized by involuntary muscle contractions leading to twisting movements and abnormal postures^1^. It can arise from childhood, adolescence and adulthood, affecting different body parts. Based on the pattern of inheritance and the genetic loci involved in dystonia, twenty-five inherited forms of dystonia (DYT1-DYT25) have been distinguished, of whom twenty-four loci have been mapped and fourteen are clearly identified as disease-causing genes^2^, including *TOR1A*^3^, *TAF1*^4^, *TUBB4*^5^, *GCH1*^6^, *THAP1*^7^, *MR-1*^8^, *PRRT2*^9^, *SGCE*^10^, *ATP1A3*^11^, *PRKRA*^12^, *GLUT1*^13^, *CIZ1*^14^, *ANO3*^15^ and *GNAL*^16^. Most forms of dystonia follow an autosomal dominant pattern of inheritance, while DYT2^17^, DYT16^12^ and DYT17^18^ are inherited in a pattern of autosomal recessive, and DYT3^19^ is an X-linked recessive dystonia. Clinical and genetic heterogeneity complicate the identification of pathogenic genes in dystonia by traditional linkage mapping. To further understand the genetics of dystonia, we focus on patients who did not carry a known genetic mutation in any of the dystonia-related genes.

In the present study, we applied whole exome sequencing (WES) to investigate the genetic cause of dystonia in a consanguineous family with adolescent-onset primary dystonia. Combined with intersection filtering, homozygosity mapping and identity-by-descent (IBD) filtering analysis of exome sequence data, we identified a novel homozygous missense mutation, c.156 C > A in a vacuolar protein sorting gene, *VPS16*, co-segregating with disease status in the family. To assess the biological role of the c.156 C > A homozygous mutation of *VPS16*, we generated mice with targeted mutation site of *Vps16* through CRISPR-Cas9 genome-editing approach. *Vps16* mutant mice exhibited significantly impaired motor function, suggesting that *VPS16* is a new causative gene of dystonia.

## Results

### Patients with adolescent-onset primary dystonia

**Figure 1:**
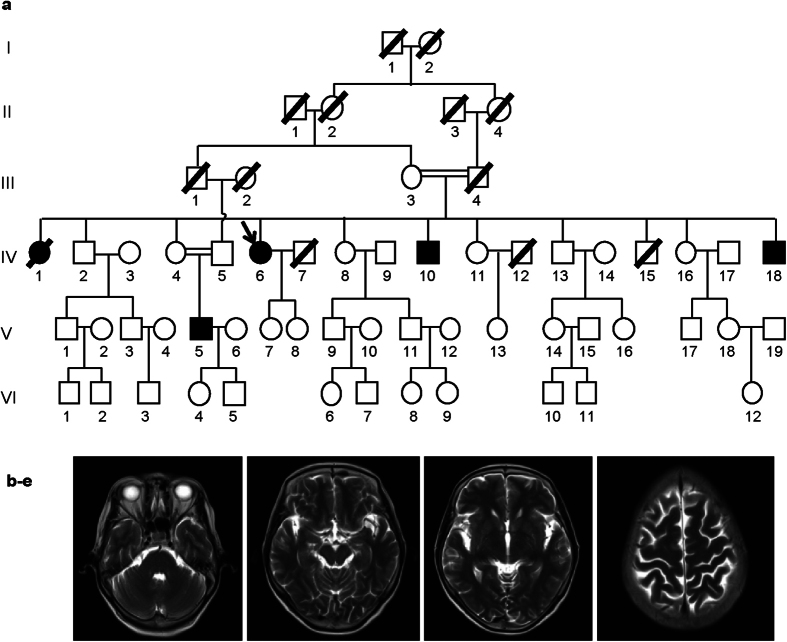
Clinical characteristics of the five patients with dystonia. Pedigree of the five patients with dystonia. Each generation is denoted by a Roman numeral, and each individual by an Arabic numeral. Empty symbols represent unaffected individuals. Solid symbols represent dystonia patients. Slashes represent deceased individuals. Double horizontal lines represent consanguious couples. The proband is indicated by arrow. (**b**) MRI image of the proband IV6.

We diagnosed a Chinese family comprising two couples of first-cousin marriage with adolescent-onset primary dystonia (Fig. 1a). Clinical features of all affected individuals are summarized in Table 1. All the affected family members developed dystonia symptom from the age of 11–14, and all began at the neck. Dystonia symptom in the proband (IV6) and two of her affected brothers (IV10, IV18) spread widely, resulting in severe motor disability; whereas patient V5 only had movement disorder in the neck. Axial cerebral T2-weighted image of the proband (IV6) showed no abnormal intracranial lesions (Fig. 1b–e).

### Genetic screening of known dystonia-related genes

To uncover the underlying genetic defect in the patients, we initially screened 24 known dystonia-related genetic loci using targeted exome-sequencing approach. Neither of them was detected in these patients, suggesting a novel genetic defect is responsible for the dystonia symptom in the family.

### Whole Exome-sequencing detects a missense variant in *VPS16*

**Figure 2:**
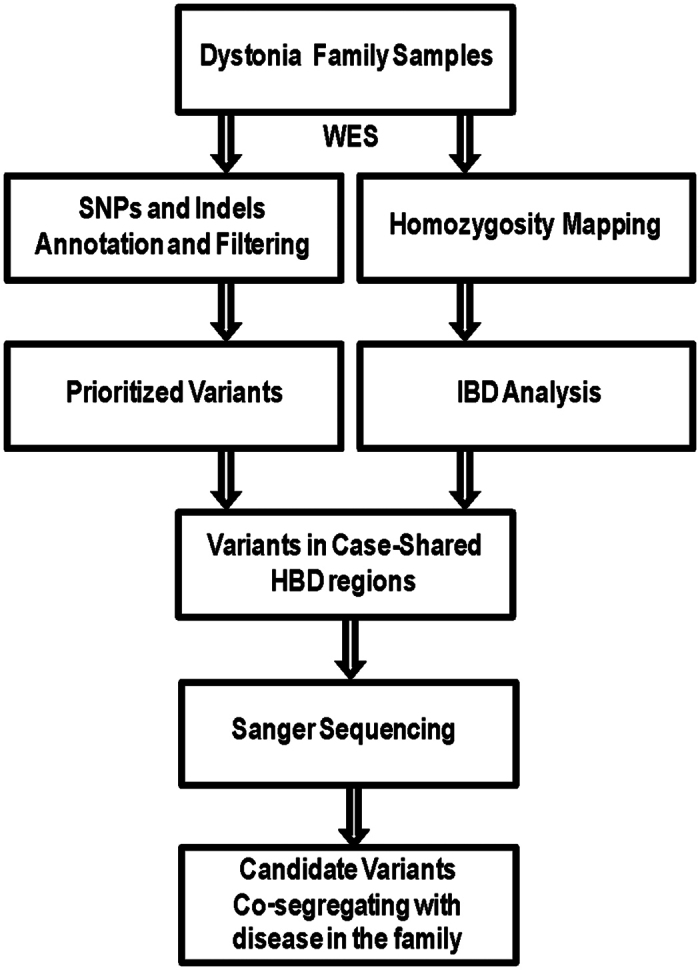
Flowchart of disease-causing gene exploration of dystonia patients from WES.

To uncover the underlying genetic defect in those patients, we sequenced the whole exomes of four affected individuals and two unaffected parents. Variants annotation and filtering analysis combined with homozygous mapping and IBD analysis from WES were used for screening candidate dystonia-causative variant in the family (Fig. 2). The study was approved by the Ethics Review Committee (ERC) of Shenzhen University 1^st^ Affiliated Hospital and BGI. WES produced approximately 100 million paired reads per sample, more than 99% of which were mapped to target region (supplementary Table S1). The average sequencing depth was 74.98X, with more than 85% reads covered > = 20X. Based on the assumption that variants that are common in the population are not likely to be the genetic cause of rare Mendelian diseases, such variants that were reported in the 1000 Genomes, dbSNP, HapMap, and YH databases with high frequency (>0.5%) are initially filtered out from our analysis (supplementary Table S2). Those rare variants including 9 SNPs and 7 Indels that were shared in all cases and absent in controls were retained as prioritized candidate dystonia-causative variant (supplementary Table S3).

**Figure 3:**
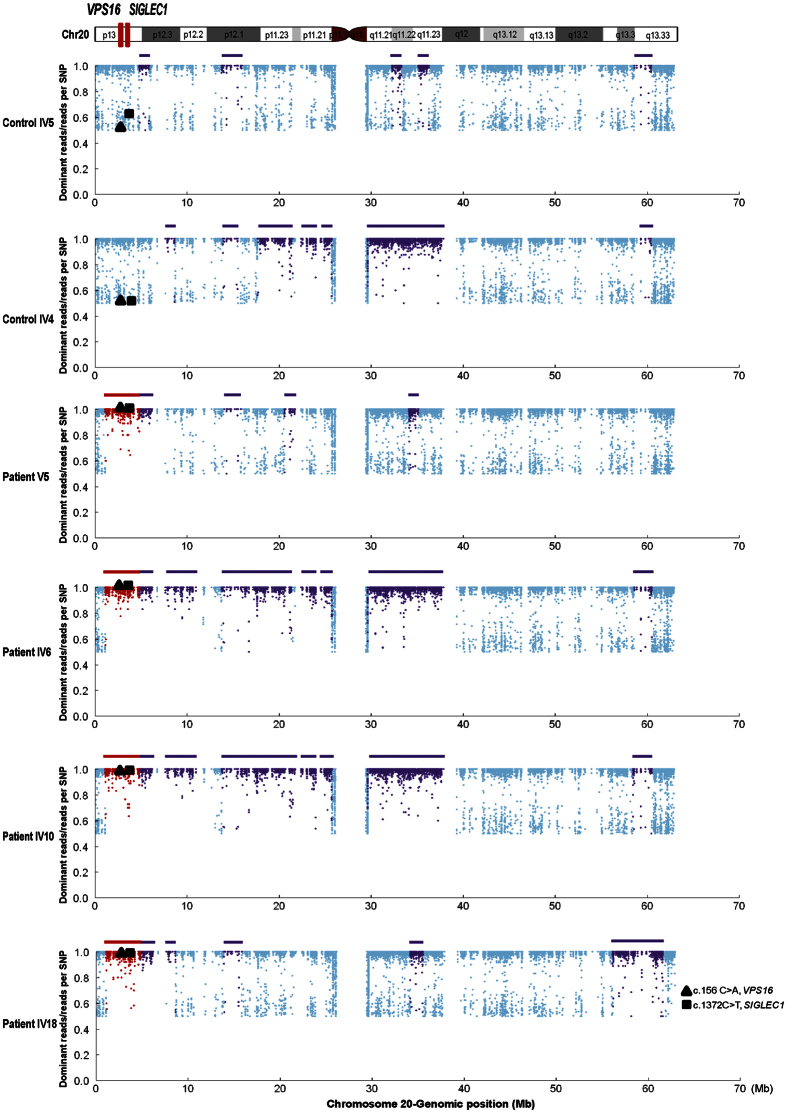
Distribution of identified homozygous region on chromosome 20. Upper panel illustrates the localizations of *VPS16* and *SIGLEC1* on chromosome 20 (red bars). Bottom panel represents the homozygous regions on chromosome 20 identified from indicated family members. Each dot represents each SNP markers identified from WES. Purple color represents SNPs within a homozygous region, whereas red represents in a case-shared and control-absent HBD. X axis indicates the genomic location of SNP markers, whereas Y axis indicates the ratio of the number of dominant reads to the total number of reads per SNP position. Triangles and squares represents novel variants (c.156 C > A; c.1372 C > T) identified in *VPS16* and *SIGLEC1* respectively.

Since the proband (IV6) and two of her affected brothers (IV10, IV18) were offspring of first-cousin marriage, as well as one of her affected nephew (V5), suggesting an autosomal recessive inheritance of the disease in this family. Therefore, the dystonia-causative variant must be located in a case-shared homozygous IBD region [homozygosity-by-descent (HBD)] and having no overlap with the controls. Based on these criteria, three case-shared HBD regions identified from homozygosity mapping and IBD analysis were retained as candidate disease-causing genetic loci (supplementary Table S4): Chr1: 178006795- 179040929 bp, Chr2: 42722370- 42809031 bp, and Chr20: 1126746- 4842635 bp (hg19; UCSC genome browser). Combined with variants filtering analysis we did in the first step, only two candidate recessive variants were found to be located in one of the HBD region on chromosome 20 (Fig. 3 and Supplementary Table S3): c.156 C > A (p.Asn52Lys) in *VPS16* (Refseq accession number NM_022575.3) and c.1372C > T (p.Asp458Asn) in *SIGLEC1* (Refseq accession number NM_023068.3).

**Figure 4:**
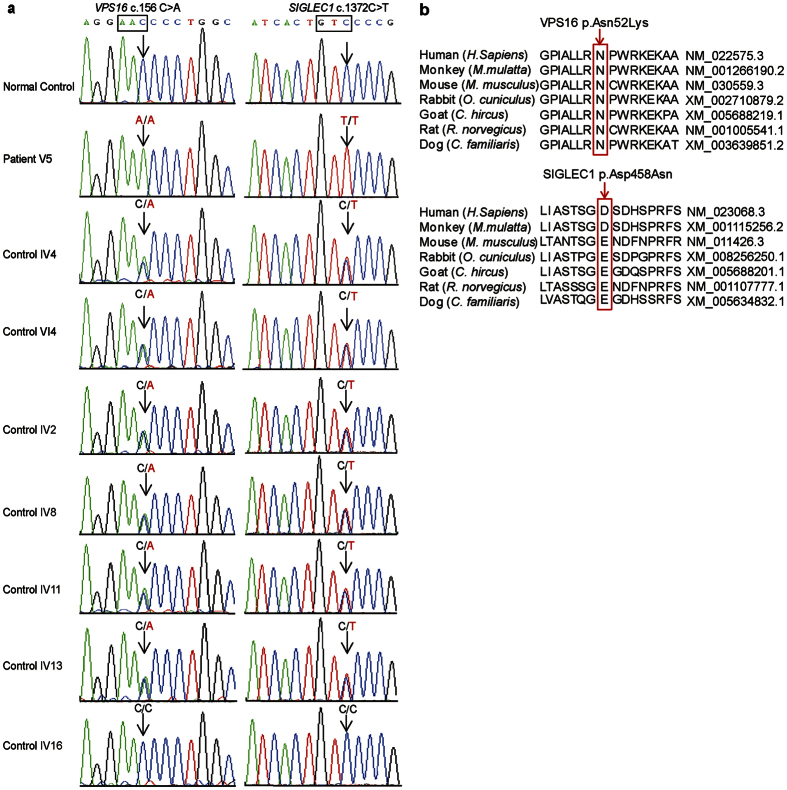
Sanger sequencing and protein conservation analysis of *VPS16* and *SIGLEC1* variants. (**a**) Sequencing chromatograms are shown. Affected codons are framed in black, and variants found in individuals are indicated by arrows. (**b**) Protein alignment of VPS16 and SIGLEC1 variants from seven different vertebrate species using ClustalW. Altered Amino acid are indicated on the top panel, whereas Refseq residues are framed in red and indicated by arrows.

Both nucleotide substitutions in *VPS16* and *SIGLEC1* were confirmed in patient V5 by Sanger sequencing (Fig. 4a). His mother (IV4) and his daughter (VI4) with normal phenotype both carried heterozygous mutations in *VPS16* and *SIGLEC1*, whereas V5 was homozygous for both mutations. And the familial segregation of both variants was consistent with disease status, showing an autosomal recessive inheritance in the family (Fig. 4a).

*VPS16*, encodes a core subunit of VPS-C complex that is required for vesicle transport and fusion process in the late endosomes/lysosomes pathway^20^,^21^. Importantly, VPS-C complex was found highly enriched in the brain and nervous system^22^, and interacted with syntaxin 1A and neuron specific SNARE proteins regulating pre-synaptic release^23^. The replacement of an Asn residue in the β-propeller domain of VPS16 with a Lys residue is highly conserved, and the homozygous missense mutation, c.156 C > A in VPS16, is predicted to have damaging impact on protein function through MutationTaster prediction^24^, supporting the pathological role of amino acid substitution (Fig. 4b, supplementary Table S3). Therefore, we highly speculated a causative role of *VPS16* gene mutation in the familial adolescent-onset primary dystonia.

The other candidate gene, *SIGLEC1* encodes an immunoglobulin superfamily protein with proinflammatory functions in macrophages^25^. We didn’t pursue it further in our study, because wide-type Asp458 of SIGLEC1 is not evolutionarily conserved in vertebrate (Fig. 4b and supplementary Table S3) and *SIGLEC1*-deficient mice only showed a minimal phenotype of immune system^26^. Therefore, based on its expression pattern and reported biological function, we considered that c.1372C > T substitution in *SIGLEC1* is an irrelevant polymorphism.

### Mutational screening of *VPS16* in 200 normal controls

Given that dystonia only affects small numbers of people, it is unlikely the genetic defect would exist in the normal people. Therefore, we screened the c.156 C > A variant of *VPS16* in 200 controls from individuals with the same geographic ancestry as the consanguineous family. No homozygous mutation in *VPS16* was detected in 200 controls (Fig. 4a), indicating it is a rare polymorphism in normal cohort.

### Mutational screening of *VPS16* in 14 sporadic dystonia patients

To examine whether *VPS16* was associated with dystonia in additional patients, we performed Sanger sequencing of the entire *VPS16* exons in 14 unrelated sporadic cases with adolescent-onset dystonia compatible with autosomal recessive inheritance. However, novel or known rare (frequency <1%) homozygous or compound heterozygous variants of *VPS16* were not detected in any of these patients, as were protein-disrupting heterozygous variants, suggesting refinement of this phenotype requires further genetic screening in additional familial and sporadic dystonia cohorts.

### Generation of *Vps16* c.156 C > A mutation mice through CRISPR-Cas9 genome-editing approach

**Figure 5:**
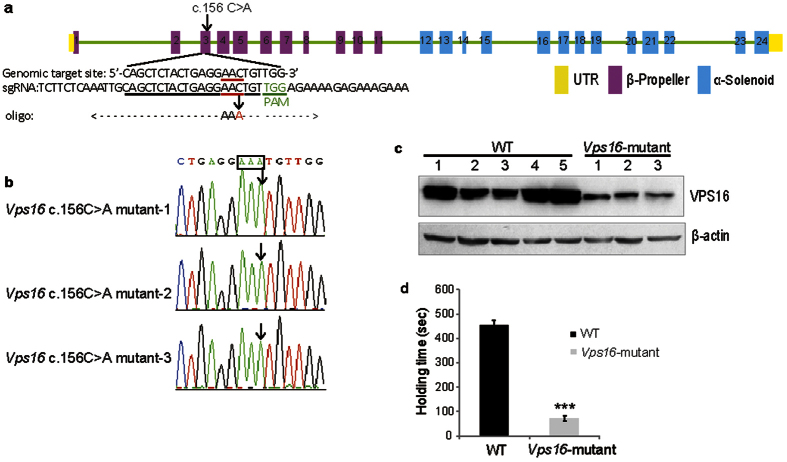
Targeted mutation of mouse *Vps16* gene. (**a**) Schematic representation of the genome localization of c.156 C > A mutation in *Vps16* gene and construction of the targeting sgRNA and oligonucleotide. UTR regions are colored in yellow, β-Propeller domain in purple and α-Solenoid domain in blue. Mutation site is indicated by red color and black arrows. PAM motif is indicated in green. (**b**) Sequencing chromatograms of *Vps16* c.156 C > A mutant mice. Mutation site is indicated by arrows. (**c**) Western blot analysis of VPS16 expression from retro-orbital blood of WT and *Vps16*-mutant mice. (**d**) Rotating rod analysis of motor function in WT and *Vps16*-mutant mice. Graph represents the mean ± SE obtained from at least three independent experiments. The unpaired Student’s T-test revealed a significant difference between WT (n = 5) and Vps16-mutant mice (n = 3). ***Represents p < 0.0001 compared to WT mice.

To further investigate the pathological role of VPS16, we generated mice with targeted c.156 C > A mutation of *Vps16* through CRISPR-Cas9 genome-editing approach. The mouse *Vps16* (NM_030559.3) shows 97% identity with the human sequence (NM_022575.3). All animal experiments were approved by the life ethics and biological safety review committee of BGI (Shenzhen, China). A single-guild RNA (sgRNA) targeting the sequence to be mutated (Fig. 5a) was coinjected along with Cas9 mRNA and a single stranded oligonucleotide (Fig. 5a) containing the c.156 C > A mutation of *Vps16* into one-cell stage C57BL/6J embryos. Following selective breeding of the *Vps16* mutant mice, we successfully generated three mice that carried homozygous c.156 C > A mutations of *Vps16*. Successful HDR-mediated mutation was confirmed by Sanger sequencing (Fig. 5b).

### Phenotype Analysis of *Vp*s16 c.156 C > A mutant mice

*Vps16* c.156 C > A mutant mice were indistinguishable from wild-type (WT) at birth, showing no obvious abnormality in the body size and weight. However, homozygous c.156 C > A mutation of *Vps16* is leading to a reduction of VPS16 protein expression in mice (Fig. 5c). Moreover, when hung by their tails at 6 month-old, *Vps16* mutant mice stayed almost still with their unbalanced hind limbs overstretched, showing obvious abnormality in the behavior compared to WT (supplementary video 1). To assess the motor balance and coordination, *Vps16* mutant and WT mice were tested by the accelerating Rotarod analysis. Three *Vps16* mutant mice all showed a significantly reduced latency to fall from the Rotarod compared to WT mice at 6 months of age (p < 0.0001), suggesting the impaired motor function (Fig. 5d, and supplementary video 2).

## Discussion

In the past decade, the application of WES has facilitated the discovery of many causative genes of mendelian disorders in pedigree-based genetic mapping. Herein, our study successfully demonstrates the identification of a novel disease-causing gene *VPS16* in a Chinese consanguineous family with adolescent-onset primary dystonia based on WES approach. The proband (IV6) and two of her affected brothers (IV10, IV18) are the offspring of first-cousin consanguineous marriage, as well as one of her affected nephew (V5), suggesting an autosomal recessive inheritance of the disease in this pedigree (Fig. 1a).

Combined with variants filtering analysis, homozygous mapping and IBD analysis from WES, a homozygous missense mutation, c.156 C > A of *VPS16*, was identified as the candidate dystonia-causative variant. Sanger sequencing confirms that the familial segregation of *VPS16* variant was consistent with disease status, showing an autosomal recessive inheritance in the family (Fig. 4a).

The frequency of *Vps16* c.156 C > A allele mutation has been reported to be 0.0029 in the East Asian population from the ExAc database (http://exac.broadinstitute.org/variant/20-2840713-C-A), whereas the homozygotes has not yet been reported. Moreover, among the 200 normal controls we screened, neither heterozygous nor homozygous mutation of *VPS16* is detected (Fig. 4a), indicating it is a rare polymorphism in the normal cohort. So far, we have not identified mutations in *VPS16* in additional unrelated patients, possibly due to limited patients we investigated. Therefore, refinement of this phenotype requires further genetic screening in additional familial and sporadic dystonia cohorts.

*VPS16* has been reported to be enriched in the brain and nervous system and co-localize with syntaxin-1 in the neuronal processes and axonal outgrowths^22^. However, it has not yet been reported to be related to any diseases. Interestingly, loss of one of its major interactors, *VPS18*, led to widespread neurogeneration through obstructing endosomal maturation, lysosomal trafficking and autophagosome clearance^27^. Therefore, we highly speculated a causative role of *VPS16* gene mutation in the familial adolescent-onset primary dystonia. The replacement of an Asn residue in the β-propeller domain of VPS16 with a Lys residue is predicted to have damaging impact on protein function through MutationTaster prediction^24^, supporting the pathological role of amino acid substitution (Fig. 4b, supplementary Table S3).

It would be interesting to mutate *Vps16* to assess its biological function. With the efficiency and ease of the CRISPR-Cas9 genome editing approach^28^,^29^, we have demonstrated how it can be applied to investing disease-causing gene in dystonia (Fig. 5a,b). Targeted c.156 C > A mutation of *Vps16* led to downregulation of VPS16 expression in the mice (Fig. 5c). Moreover, *Vps16* mutant mice exhibited obvious abnormality in the behavior and significantly impaired motor function (Fig. 5d, supplementary video 1 and 2), indicating that *VPS16* is the genetic cause of familial adolescent-onset primary dystonia.

To our knowledge, this is the first demonstration that the mutation of *VPS16* was identified in cases of adolescent-onset primary dystonia. The functional characterization of targeted *Vps16* c.156 C > A homozygous mutation in mice also supports its disease-causing role in dystonia, thus providing new insights into the pathogenesis of this common movement disorder.

## Methods

### Clinical evaluations

The family with adolescent-onset primary dystonia was ascertained from Hunan province. Human studies were conducted in accordance with the Declaration of Helsinki, with formal approval from the Ethics Reviewing Committee of Shenzhen University 1^st^ Affiliated Hospital and BGI. All related experiments were carried out only after written informed consent was obtained from each individual and/or parents of the children. The medical history was obtained by use of a questionnaire regarding the following aspects: age at onset, set of onset and evolution. The Burke-Fahn-Marsden dystonia rating scale (BFMDRS) was applied for motor assessment. The motor score consists of a scale assess movement impairment of the speech, eyes, trunk and extremities^30^. The movement assessment was performed by a specialized neurologist. MRI images were acquired from patient IV6 on a 1.5 Tesla Siemens scanner to display the brain image (SIEMENS AG, Munich, Germany).

### Exome capture and sequencing

Genomic DNA was extracted from peripheral blood samples using DNeasy Blood and Tissue kit (Qiagen, Hilden, Germany). Exome capture and sequencing were performed in BGI according to the manufacturer’s protocol. Briefly, genomic DNA was randomly sonicated to generate short fragments between 250 bp and 300 bp. Then adaptors were subsequently added to ligate each ends of the fragments. After amplified by ligation-mediated PCR, the pooled DNA fragments were hybridized to NimbleGen SeqCap EZ Exome (64 M) capture array (Roche NimbleGen Inc, Wisconsin, USA) for target enrichment, followed by washing and amplification. The captured DNA libraries were then sequenced on a Hiseq 2000 platform (Illumina, San Diego, USA).

### Read mapping and variant analysis

The sequencing reads were analyzed as previously described^31^. The raw image files were initially processed using the Illumina pipeline software (version 1.7) for base calling, and generated 90-bp paired-end reads. Clean reads were then mapped to the reference human genome (GRCh37/hg19) from UCSC genome browser (http://genome.ucsc.edu/) with SOAPalinger software (http://soap.genomics.org.cn/index.html). SNPs were then detected by using SOAPsnp software (http://soap.genomics.org.cn/index.html). Indels were aligned to the UCSC reference human genome using BWA software (http://bio-bwa.sourceforge.net/) and further processed with the Genome Analysis Toolkit (GATK v1.6) for recalling. SNP calls and Indels were filtered to coordinate with the following criteria: 1) consensus quality score ≥20; 2) sequencing depth ≥4 and ≤500; 3) copy number ≤2; 4) distance between two adjacent SNPs ≥5 bp.Variants in non-coding region and synonymous mutations were removed from our analysis. Non-synonymous mutations, frame-shifting Indels, splice acceptor and donor site mutations that could potentially affect normal protein functions were retained. Common SNPs and Indels presented in dbSNP (http://www.ncbi.nlm.nih.gov/projects/SNP/, build 137), 1000 Genomes (ftp://www.1000genome.org), HapMap and YH database with high frequency (>0.5%) were also filtered out. Novel variants that were presented as homozygous call in at least one case were considered as prioritized candidate variants. Given that all the affected individuals were related, only the recessive alleles that presented in the homozygous region were retained as disease-causing candidate variant for Sanger validation. Candidate variants were analyzed using SIFT^32^, Polyphen-2^33^ and Mutation Taster^24^ software to determine possible changes in protein structure that could affect phenotype.

### Exome Homozygosity mapping

The data from WES were used for examining large stretches of homozygous region as previously reported^34^,^35^. All the autosomal dbSNP sites and novel SNPs that had ≥20-fold coverage of the exome target regions were selected as markers to create a genetic map. Variants displaying an identical SNP allele with ≥95% of all reads and covering at least 5-fold were considered to be homozygous markers. SNPs with 30–70% variation reads and covered at least 10-fold were considered to be heterozygous markers. And SNPs with <30% or with 70–95% variation reads were considered ambiguous. Statistics on the distribution of map markers along the genome were analyzed with Perl scripts. We adopted a window of 500 markers, containing maximum two heterozygous markers and allowing the gap between two adjacent markers was maximum 500 Kb. A genomic region was identified as a homozygous stretch as coalescence of all qualified windows with minimum of 1 Mb in length^36^.

### Sanger sequencing

Candidate variants in *VPS16* and *SIGLEC1* were validated by Sanger sequencing of DNA isolated from the family with dystonia and 200 randomly selected normal controls using primers as follows:

*VPS16* F: 5′-GAAGACAAATGGAGGCTTTAGG-3′,

*VPS16* R: 5′-ACTGCAGGGGAGAGAGGTTC-3′

*SIGLEC1* F: 5′- GCACTCAGGCACTTTGGG-3′

*SIGLEC1* R: 5′- GTGGCGTAGCCAGTTAGCTC-3′

Primers used for sequencing whole exome of *VPS16* are listed as follows:

Exon 1 F: 5′-CTGCCCACAGTGGTGATG-3′

Exon 1 R: 5′-CAGTAGTGACAGGGGCGAAC-3′

Exon 2–6 F: 5′- GCCTTGTGGAAGACAAATGG

Exon 2–6 R: 5′- GGTAGGGGTGGGACAGATAC

Exon 7–11 F: 5′- CCCTGAGTGGGAATGAAGTG-3′

Exon 7–11 R: 5′-CTGCCTGGACCCCTCTC-3′

Exon 12–15 F: 5′CCAACCCAGCTTATTTGAACC-3′

Exon 12–15 R: 5′- GCCCAGCGCAGTTCTACAC -3′

Exon 16–20 F: 5′-AAGAAAGAAGTGGCGGGG-3′

Exon 16–20 R: 5′-TTGATCCTCTGTAGCCTGGG-3′

Exon 21–23 F: 5′-GAGTTTGCAGCCAAGGTTTG-3′

Exon 21–23 R: 5′-AAGAGAGGAGGTGGTGTTGG-3′

Exon 24 F: 5′-GTCAACTGAGGGCCTGTGG-3′

Exon 24 R: 5′-GCATTGCTCTAGGGGAGGAG-3′

### Pairwise IBD segments estimation

Plink software^37^ (http://pngu.mgh.harvard.edu/~purcell/plink/contact.shtml#cite) (v1.06) was used for pairwise IBD estimation to find pairs of individuals who look too similar to each other. The threshold limit of IBD segments was defined as a stretch of runs of at least 50 consecutive homozygous SNPs encompassing a minimum of 1 Mb. Remaining options were set to default values.

### Animal use and care

All animal procedures were approved by the life ethics and biological safety review committee of BGI, and were carried out in accordance with the approved guidelines.

### CRISPR vector and single guide RNA (sgRNA) cloning

To construct the mammalian Cas9 expression vector, the sequence of Streptococcus pyogenes Cas9 gene was codon-optimized for expression in mammalian cells. A T7 promoter sequence amplified from pcDNA3.1 plasmid was subcloned on the upstream of Cas9 cDNA sequence, allowing for *in-vitro* Cas9 mRNA synthesis. The nuclear localization sequence of SV40 large T-antigen was added at the 5′ and 3′ end of Cas9 gene. The DNA oligo pairs for expressing sgRNA were ordered from Generay Biotech Company (Shanghai, China):

F: 5′- ACCGAGCTCTACTGAGGAACTGTG-3′

R: 5′- AAAACACAGTTCCTCAGTAGAGCT-3′

The two complementary oligos were denatured at 95 °C for 5 minutes, ramp cooled to 25 °C over a period of 45 min to allow annealing, and finally ligated with the linearized pX458. A single stranded oligonucleotide containing the desired mutation in the targeted region of *Vps16* was designed and ordered. The oligonucleotide sequence was as follows:

5′-GGTTCTAGGACAGAAGTCTCTTCTCAAATTGCAGCTCTACTGAGGAAATGTTGGAGAAAAGAGAAAGCTGCCAGCGTGCGGCCAGTACTGGAG-3′.

### *In-vitro* transcribing Cas9 mRNAs and sgRNAs

Linearized Cas9 plasmids by BamHI enzyme (NEB, USA) were used as templates for Cas9 mRNA synthesis via *in-vitro* transcription by using mMESSAGE mMACHINE T3 kit (Life technologies, USA). Newly synthesized Cas9 RNA was subsequently 3′-end polyA tail by polyA polymerase tailing kit (Epicentre, USA). sgRNAs were amplified using primer:

F: 5′-TAATACGACTCACTATAGGACCGAGCTCTACTGAGGAACTGTG-3′

R: 5′-AAAAAAGCACCGACTCGGT-3

And then *in-vitro* transcribed using mMESSAGE mMACHINE T3 kit.

### Microinjection of zygotes

C57BL/6J mice were purchased from the Animal Center of Guangdong Medical Laboratory (Guangzhou, China). Eight to ten week-old female mice were superovulated by injection with 10 IU of equine chorionic gonadotrpin and HCG (Ningbo second hormone factory, Ningbo, China) and mated with 8–10 week-old males. One-cell stage embryos were collected for microinjection of Cas9 mRNAs (100 ng/μl), sgRNA (50 ng/μl) and oligonucleotide (100 ng/μL), using a standard microinjection system (Eppendorf, Hamburg, Germany). The embryos were then cultured in M2 medium (Omega, USA), and immediately after turning into the blastocyst stage, the embryos were transferred into pesudopregnant female mice.

### Sanger sequencing of *Vps16* mutant mice

Genomic DNA was prepared from the tails of three-week-old mice and CRISPR/Cas9-induced *Vps16* c.156 C > A mutations were identified through Sanger sequencing. The region including CRISPR/Cas9 target site was PCR-amplified by using primer as follow:

*Vps16* F: 5′-ATCCCCCTCTCTTCCTCTCAT-3′

*Vps16* R: 5′-AGGCTCCTGTGGCTCCTACA-3′

### Western Immunoblot analysis

Retro-orbital blood samples were collected from the right retro-orbital plexus of anesthetized mice as described^38^. Anesthesia was induced by placing each mouse in an inhalation chamber with 4% isoflurane (RWD Life Science, San Diego, USA) regulated with a calibrated vaporizer. For each mouse approximately 0.5 ml of heparin-anticoagulated blood was collected. Whole blood samples were incubated for 10 min with erythrocyte lysis buffer and centrifuged at 1500 rpm for 10 min. For western blotting, harvested cells were lysed in RIPA buffer. After determining the protein content of the cell lysates, the protein extracts were separated by 10% SDS-PAGE, transferred to a PVDF membrane and incubated with primary antibody (VPS16, β-actin antibodies were purchased from Santa Cruz Biotechnology, Inc., Texas, USA). The signal was detected by ECL detection system (GE Healthcare, USA).

### Rotarod test

The rotarod equipment (YLS-4C, Jinan Yiyan Scientific Research Company, Shandong, China) was used to examine the motor balance, coordination and strength of mice. Six-month old mice were trained three times for three consecutive days at 30 rpm per day. On the fourth day, WT mice and *Vps16*-mutant mice underwent testing. The latency to fall from the Rotarod apparatus was recorded automatically and analyzed later. The experiment was repeated at least three times.

### Data analysis

All data are the mean ± SE for at least 3 experiments. Data were analyzed statistically using the unpaired Student’s t test. The criterion for a significant difference was ***p < 0.0001.

## Additional Information

**How to cite this article**: Cai, X. *et al*. Homozygous mutation of *VPS16* gene is responsible for an autosomal recessive adolescent-onset primary dystonia. *Sci. Rep.* **6**, 25834; doi: 10.1038/srep25834 (2016).

## Supplementary Material

Supplementary Information

Supplementary video 1

Supplementary video 2

## Figures and Tables

**Table 1:** Clinical characteristics of five individuals with *VPS16* mutations in the family.

Pedigree No	Gender	Age onset (y)	Age exam (y)	Sites involved											Dystonia distribution	Site of onset	Fahn- Marsden Rating Scale
				Face	Neck	Larynx	Pharynx	Tongue	Jaw	Right arm	Left arm	Right leg	Left leg	Trunk			
IV1	F	11	Dead	**·**	**·**	**·**	**·**	**·**	**·**	**·**	**·**	**·**	**·**	**·**	G	Neck	–
IV6	F	12	59	**·**	**·**	**·**	**·**	**·**	**·**				**·**	**·**	G	Neck	36
IV10	M	13	58	**·**	**·**	**·**	**·**	**·**	**·**	**·**	**·**	**·**	**·**	**·**	G	Neck	74
IV18	M	13	45	**·**	**·**	**·**	**·**	**·**	**·**	**·**	**·**	**·**	**·**	**·**	G	Neck	44
V5	M	14	35		**·**										F	Neck	2

Gender: F, female; M, male. Dystonia distribution: G, generalized; F, focal.
