# Supplementary material for: Homozygous mutation of VPS16 gene is responsible for an autosomal recessive adolescent-onset primary dystonia
Source: Sci Rep. 2016 May 12;6:25834. doi: 10.1038/srep25834 (PMC4865952; doi:10.1038/srep25834)
Supplement: Supplementary Information [file srep25834-s1.doc]

**Supplementary information**

**Homozygous mutation of *VPS16* gene is responsible for an autosomal recessive adolescent-onset primary dystonia**

Xiaodong Cai^1+^; Xin Chen^2,3+^; Song Wu^4+^; Wenlan Liu^1,5+^; Xiejun Zhang^1,6+^; Doudou Zhang^1^; Sijie He^3^; Bo Wang^1,6^; Mali Zhang^1^; Yuan Zhang^5^; Zongyang Li^1^; Kun Luo^7^，Zhiming Cai^4^ & Weiping Li^1*^

^1^Department of Neurosurgery, Shenzhen Second People’s Hospital, Shenzhen University 1^st^ Affiliated Hospital, Shenzhen, Guangdong 518035, China;

^2^Department of Urology, Sun Yat-sen University Cancer Center;  State Key Laboratory of Oncology in South China; Collaborative Innovation Center for Cancer Medicine, Guangzhou 510060, China;

^3^BGI Cognitive Genomics Lab, Building No. 11, Beishan Industrial Zone, Yantian District, Shenzhen, Guangdong518083, China;

^4^Collaborative Innovation Center for Cancer Medicine National-Regional Key Technology Engineering Laboratory for Clinical Application of Cancer Genomics, Shenzhen Second People’s Hospital, Shenzhen University 1^st^Affiliated Hospital, Shenzhen, Guangdong 518035, China;

^5^The Central Laboratory, Shenzhen Second People’s Hospital, Shenzhen University 1^st^ Affiliated Hospital, Shenzhen, Guangdong 518035, China;

^6^The First Clinical College of Anhui Medical University, Hefei 230032, China;

^7^School of Medicine, Shandong University, 44 West Culture Road, Ji'nan, Shandong 250012, China

^*^Correspondence should be addressed to Dr. Weiping Li, Department of Neurosurgery, Shenzhen Second People’s Hospital, Shenzhen University 1^st^ Affiliated Hospital, Shenzhen, Guangdong 518035, China; [Tel:+86-755-83368128](tel:+86-755-83368128), Fax: +86-755-83356952, Email: liweiping60@126.com.

^+^The authors contributed equally to this work.

**Supplementary Table S1: Summarized sequencing metrics of WES from the family with dystonia**

| Detail Information/ Sample | Control IV4 | Control IV5 | Case IV6 | Case IV10 | Case IV18 | Case V5 | Mean |
| --- | --- | --- | --- | --- | --- | --- | --- |
| Raw reads | 100863356 | 98022404 | 99472432 | 104993054 | 102976112 | 90302258 | 99438269 |
| Raw data yield (Gbp) | 9.08 | 8.82 | 8.95 | 9.45 | 9.27 | 8.13 | 8.95 |
| % Reads mapped to genome | 87.10 | 88.53 | 88.44 | 87.64 | 86.40 | 82.12 | 86.70 |
| % Reads mapped to target region | 54.76 | 55.23 | 54.32 | 54.41 | 53.77 | 51.50 | 54.00 |
| Mean depth of target region (X) | 77.26 | 75.45 | 75.58 | 79.61 | 77.17 | 64.81 | 74.98 |
| Coverage of target region (%) | 99.16 | 99.10 | 99.15 | 99.14 | 99.20 | 99.11 | 99.14 |
| Fraction of target corvered >=20X (%) | 93.72 | 93.20 | 93.22 | 93.53 | 91.74 | 86.50 | 91.99 |

**Supplementary Table S2: Annotation of variants identified in WES from the family with dystonia**

| Detail information (SNPs and Indels) / Sample | Control II4 | Control II5 | Case II6 | Case II10 | Case II18 | Case III5 |
| --- | --- | --- | --- | --- | --- | --- |
| Total | 133842+10985 | 137049+11166 | 136694+10891 | 133258+10812 | 134279+10821 | 125231+10107 |
| Protein-disrupting SNPs and Indels (PDSI) | 15484+1700 | 15721+1732 | 15491+1746 | 15013+1733 | 15420+1753 | 15230+1633 |
| PDSI after filtered against dbSNP | 2272+514 | 2258+509 | 2313+525 | 2181+518 | 2229+535 | 2198+470 |
| PDSI after filtered against dbSNP+1000Genomes | 1548+286 | 1523+289 | 1553+294 | 1445+305 | 1525+299 | 1472+282 |
| PDSI after filtered against dbSNP+1000Genomes+HapMap | 1546+286 | 1523+289 | 1551+294 | 1445+305 | 1525+299 | 1470+282 |
| PDSI after filtered against dbSNP+1000Genomes+HapMap+YH | 1511+285 | 1496+288 | 1518+293 | 1416+303 | 1491+297 | 1448+280 |
| Prioritized variants that shared in all cases and absent in controls | - - 9+7 9+7 9+7 9+7 | | | | | |
| Presented in case-shared HBD region | - - 2+0 2+0 2+0 2+0 | | | | | |

**Supplementary Table S3: Summary of prioritized variants that are shared in all cases and absent in controls**

| Chr | Position | Gene | Mutation Type | Ref | Alt | Substitution | SIFT  (<0.05) | PolyPhen2_  HDIV(>0.452) | MutationTaster (Prediction) |
| --- | --- | --- | --- | --- | --- | --- | --- | --- | --- |
| chr3 | 108754320 | MORC1 | Indel | T | +AA |  | - | - | - |
| chr5 | 137507105 | BRD8 | Indel | A | -A |  | - | - | - |
| chr8 | 76468310 | HNF4G | Indel | T | -TT |  | - | - | - |
| chr12 | 133195580 | P2RX2 | Indel | G | -GCGGGGCGCGGGGT |  | - | - | - |
| chr13 | 77835520 | MYCBP2 | Indel | T | +A |  | - | - | - |
| chr13 | 113818817 | PROZ | Indel | T | +C |  | - | - | - |
| chr19 | 46273463 | DMPK | Indel | C | -CAGCAGCAGCAGCAGCAGCAGCAGCAGCAGCAGCAGCAGCAGCAG |  | - | - | - |
| chr7 | 143885454 | ARHGEF35 | SNP | T | C | H8R | 0.151 | 0.995 | Polymorphism |
| chr8 | 7673126 | DEFB107A | SNP | C | A | V9F | 1 | - | Polymorphism |
| chr8 | 145757732 | ARHGAP39 | SNP | G | C | D979E | 0.093 | 0.998 | Disease_causing |
| chr9 | 43822762 | CNTNAP3B | SNP | C | T | P439L | 0.791 | - | Polymorphism |
| chr10 | 51363294 | PARG | SNP | T | C | K260E | - | - | - |
| chr19 | 54745682 | LILRA6 | SNP | C | T | R143Q | 1 | 0.393 | Polymorphism |
| chr20 | 2840713 | VPS16 | SNP | C | A | N52K | 0.084 | 0.135 | Disease_causing |
| chr20 | 3682145 | SIGLEC1 | SNP | C | T | D458N | 0.015 | 0.409 | Polymorphism |
| chrX | 70937004 | CXorf49B | SNP | A | G | L333P | - | - | - |

**Supplementary Table S4: Nine case-shared and control-absent homozygous regions were identified from homozygosity mapping**

| Chromosome | Start Position (hg19) | End Position (hg19) | Size (Mb) | IBD sharing events among case/case pairing | Genes |
| --- | --- | --- | --- | --- | --- |
| Chr1 | 178006795 | 179040929 | 1.03 | 4/4 | ANGPTL1, C1orf220, FAM20B, LOC730102, MIR4424, RALGPS2, RASAL2, TEX35 |
| Chr2 | 42722370 | 42809031 | 0.08 | 4/4 | MTA3 |
| Chr2 | 109276382 | 109929957 | 0.65 | 2/4 | CCDC138, EDAR, LIMS1, MIR4265, RANBP2, SH3RF3, SH3RF3-AS1 |
| Chr2 | 222384481 | 223436427 | 1.05 | 0/4 | CCDC140, EPHA4, FARSB, PAX3, SGPP2 |
| Chr5 | 128365536 | 128441120 | 0.07 | 3/4 | ISOC1, MIR4633, SLC27A6 |
| Chr6 | 87795864 | 88366772 | 0.57 | 2/4 | C6orf162, C6ORF163, C6ORF164, C6ORF165, CGA, GJB7, ORC3, RARS2, SLC35A1, ZNF292 |
| Chr7 | 70800469 | 71252190 | 0.45 | 2/4 | CALN1, WBSCR17 |
| Chr12 | 93788281 | 94543505 | 0.75 | 2/4 | CRADD, MRPL42, NUDT4, NUDT4P1, PLXNC1, SOCS2, SOCS2-AS1, UBE2N |
| Chr20 | 1126746 | 4842635 | 3.71 | 4/4 | ADAM33, ADRA1D, AP5S1, ATRN, AVP, C20orf141, C20orf194, C20orf202, C20orf27, CDC25B, CENPB, CPXM1, DDRGK1, EBF4, FASTKD5, FKBP1A, FKBP1A-SDCBP2, GFRA4, GNRH2, HSPA12B, IDH3B, ITPA, LOC100289473, LOC100507495, LOC728228, MAVS, MIR103A2, MIR103B2, MIR1292, MRPS26, NOP56, NSFL1C, OXT, PANK2, PCED1A, PDYN, PRND, PRNP, PRNT, PROSAPIP1, PSMF1, PTPRA, RAD21L1, RASSF2, RNF24, SDCBP2, SIGLEC1, SIRPA, SIRPB1, SIRPB2, SIRPD, SIRPG, SLC23A2, SLC4A11, SMOX, SNORA51, SNORD110, SNORD119, SNORD56, SNORD57, SNORD86, SNPH, SNRPB, SPEF1, STK35,TGM3,TGM6, TMC2, TMEM239, TMEM74B, UBOX5, UBOX5-AS1, VPS16, ZNF343 |

**Supplementary Vidio 1. Tail hanging test in the *Vps16* c.156 C>A homozygous mutation mice**

**Supplementary Vidio 2. Rotarod test in the *Vps16* c.156 C>A homozygous mutation mice**
